# Is preterm donor milk better than preterm formula for very-low-birth-weight infants?

**DOI:** 10.29219/fnr.v65.5346

**Published:** 2021-09-24

**Authors:** Lingyu Fang, Meili Zhang, Lianqiang Wu, Ruiquan Wang, Bangbang Lin, Jianfeng Yao, Dongmei Chen

**Affiliations:** 1 Neonatal Intensive Care Unit, Quanzhou Women and Children’s Hospital, Quanzhou, Fujian, China; 2 Administrative Office, Quanzhou Women and Children’s Hospital, Quanzhou, Fujian, China; 3 Reproductive Medicine Center, Quanzhou Women and Children’s Hospital, Quanzhou, Fujian, China

**Keywords:** milk bank, very-low-birth-weight infant, development and growth, necrotizing enterocolitis, sepsis

## Abstract

**Background:**

Preterm human milk has advantages over preterm formula (PF), but it may compromise some functions after pasteurization.

**Objective:**

To explore the effects of preterm donor milk (DM) on growth, feeding tolerance, and severe morbidity in very-low-birth-weight infants.

**Method:**

This was a single-center, prospective cohort study that included 304 preterm infants weighing <1,500 g or of gestational age <32 weeks. If the mother’s own milk was insufficient, the parents decided to use PF (*n* = 155) or DM (*n* = 149). The two groups were uniformly managed according to the standard NICU protocol. Growth parameters, feeding tolerance, and severe morbidity such as necrotizing enterocolitis, were compared between the two groups.

**Results:**

The daily weight gain and weekly head growth in the DM group were not different from those in the PF group (*P* > 0.05). Feeding intolerance in the DM group was significantly lower than that in PF group (*P* < 0.05), and parenteral nutrition time and hospitalization time were also shorter than that in the PF group (*P* < 0.05). Moreover, the incidence of necrotizing enterocolitis and sepsis was also significantly lower in the DM group (*P* < 0.05).

**Conclusion:**

The study indicated that preterm DM does not affect the growth of very-low-birth-weight infants. Further, it significantly reduces feeding intolerance, helps achieve full enteral feeding early, and has protective effects against necrotizing enterocolitis and sepsis. Thus, compared with formula, preterm DM can lower the rate of infection in preterm infants and is worthy of promotion.

## Popular scientific summary

Donor milk may damage its nutritional components and immunological factors due to pasteurization, and its clinical effect on very-low-birth-weight infants is questioned.

In this prospective study, feeding very-low-birth-weight infants with preterm donor milk did not affect the growth, but reduced feeding intolerance, necrotizing enterocolitis, and infection.

By encouraging the mothers of the very preterm infant to donate milk, the benefits for preterm infants can be made to a greater extent.

Human milk is the first choice for all babies, especially for premature infants. A large number of studies have shown that human milk is particularly important for preterm infants, especially for very-low-birth-weight (VLBW) infants, as it can reduce the morbidities associated with preterm birth, for example, feeding intolerance, necrotizing enterocolitis (NEC), chronic lung disease, and retinopathy. Human milk can also improve the neurobehavioral prognosis of preterm newborn babies and reduce the risk of metabolic syndrome in late adulthood, such as insulin-dependent and non-insulin-dependent diabetes mellitus, overweight and obesity, and hyperlipidemia (1–3). Enteral feeding should be initiated as early as possible after birth in order to improve tolerance against enteral feeding and prevent intestinal atrophy (4). In particular, the colostrum provides strong resistance against infection.

However, the colostrum cannot be obtained in the first few days after birth in most preterm infants. Some mothers fail to provide adequate milk to meet the infants’ requirements due to various reasons, such as immature lactation function or even need to interrupt breastfeeding because of diseases. In such cases, donor milk (DM) is considered to be a good substitute for mother’s own milk (MOM).

According to the current recommendations (5, 6), fresh MOM is the first choice for preterm infants. When the MOM is not available and sufficient, appropriately screened and pasteurized DM is recommended. Only when there is neither MOM nor DM, can preterm formula (PF) be selected. However, while milk banks are common in European and American countries, most premature babies in China are mainly fed with unpasteurized MOM or PF in the neonatal intensive care unit. China’s non-profit milk banks were established only quite recently. Between March 2013 and December 2016, 14 milk banks were established in mainland China, and a total of 3,121 mothers donated milk, including 2,828 (90.6%) term mothers (7) . It is not clear whether feeding with DM, especially preterm DM after pasteurization, is as effective as breastfeeding, as there have been no large-scale controlled studies on the effectiveness of pasteurized preterm DM in China. In August 2017, we established the first all-public welfare milk bank in Fujian Province, China, for storage of DM mainly from preterm infant mothers. The present study conditionally observes the effects of preterm DM on growth, feeding, and severe morbidity in VLBW infants.

## Methods

### Design

This study was a prospective cohort study on the benefits of preterm DM for VLBW infants whose mothers cannot provide enough milk, conducted from August 2017 to October 2019. After admission, the infants were given DM or PF for supplementary feeding, or alternative feeding, according to the parents’ decision. Those who received DM were assigned to the DM group, and those who received PF were assigned to the PF group. The inclusion criteria were birth weight <1,500 g or gestational age <32 weeks, and admission within 24 h after birth. The exclusion criteria were congenital genetic metabolic disease or congenital developmental malformation (such as cyanotic heart disease and gastrointestinal malformation), surgical treatment for conditions other than NEC, and premature termination of treatment or death before completion of treatment. Additionally, in cases where the feed was composed of 50% MOM, the infants were excluded. The ethical approval for the study was obtained from the Medical Research Committee of Quanzhou Women and Children’s Hospital. The parents provided their signed informed consent for accepting DM.

*Standardized feeding regimen*: The PF group received PF, while the DM group received DM and human milk fortifier. Both groups received parenteral nutrition, and enteral feeding was considered to have been achieved when the feeding volume reached 120~130 mL/kg per day. Once enteral feeding was achieved, the deep vein catheter was removed. On the first day after birth, micro-enteral feeding was initiated. Additionally, an umbilical vein catheter (UVC) or peripherally inserted central catheter (PICC) was used to support parenteral nutrition: initial daily amino acid dose of about 2.0 g/kg (daily increase, 1.0 g/kg; maximum daily dose, 3.5~4.0 g/kg), and addition of fatty acid 24 h after birth at an initial daily dose of 1.0 g/kg (daily increase, 1.0 g/kg; maximum daily increase, 3~3.5 g/kg). According to the feeding tolerance, enteral feeding was increased by 10~20 mL/kg daily. In the DM group, when the enteral feeding volume achieved 50~100 mL/kg daily, human milk fortifier was added. Initially, only 25% of the standard amount of fortifier was added, and it was gradually increased till the full fortification dose was reached, provided no feeding intolerance occurred, within 5~7 days. In the later stage, non-nutritive sucking training and kangaroo nursing were performed. When the patient’s weight reached 2.0 kg, DM was discontinued and replaced with formula.

### DM source

The study was conducted in a large neonatal intensive care unit at the Quanzhou Women and Children’s Hospital, Fujian Province, China. In 2017, the hospital established the first milk bank in the region and exclusively collected DM from mothers of very preterm infants (mostly VLBW infants hospitalized during the same period). All mothers were previously tested to make sure they were not positive for human immunodeficiency virus, syphilis, hepatitis B, hepatitis C, and cytomegalovirus. As blood tests ideally need to be done 3~5 days before donation of milk and colostrum production is generally low, the milk sources were transitional milk and mature milk. Preterm DM was collected in a special container (a 150 mL or 80 mL bottle designed to store DM), pasteurized at 62.5°C for 30 min, and quickly cooled down to 25°C within 10 min in an ice water bath; it was then stored in a refrigerator at a temperature of 0~4°C for 1 or 2 days or frozen at −20°C for 3~6 months. Fresh DM was preferred over frozen milk. If the total amount after pasteurization exceeded the requirement for the day, the excess was directly frozen after cooling. Frozen milk was thawed before it was administered to suitable neonates within 24 h. As of August 3, 2019, we had received 1,466,218 mL of DM from 121 mothers, mean age 29 ± 5 years, and this has benefited 593 preterm infants. The first donation time was 3.4 ± 2.2 weeks postpartum (postmenstrual age 32 ± 2 weeks), and the last donation time was 9.1 ± 4.7 weeks postpartum (postmenstrual age 38 ± 4 weeks), and the duration was 32 (19,52) days. Proportion of donor pool, transitional milk was in 3.4%; mature milk was in 96.6%; fresh milk was in 61.4%, of which 12.5% was used within 1–2 h; and frozen milk was in 38.6%, with frozen time for 0–10 days, 10–20 days, 20–30 days, 30–60 days, and 60–90 days accounted for 13.3%, 50.6%, 14.2%, 16.1%, and 5.8%, respectively

### Data collection

General information about the infants, including gestational age, gender, weight, head circumference, Apgar score, and steroid application during delivery, was obtained. The measurement objects included growth indicators and morbidity, as follows:

1. The growth indicators were weight and head circumference at discharge, average daily weight gain, weekly head circumference growth, and duration of parenteral nutrition (or placement of the central venous catheter).
2. The signs of feeding intolerance are (1) bloating or vomiting, (2) gastric retention ≥ 30% of the feeding amount, (3) obvious bloody stool, (4) bile reflux or vomiting, and (5) presence of blood-tinged or hemorrhagic substance in the stomach. If these signs were noted, feeding was discontinued.
3. The following outcomes were recorded: NEC, defined as Bell stage II and above; retinopathy of prematurity (ROP), defined as ROP stage III or higher; severe intraventricular hemorrhage (IVH), defined as stage III–IV IVH and periventricular leukomalacia (PVL). Sepsis was defined as late-onset sepsis and included clinical sepsis with negative blood culture. Bronchopulmonary dysplasia (BPD) was defined as any oxygen dependence (FiO^2^ > 21%) exceeding 28 days (or after postconceptional 36 weeks).

### Data analysis

After the data collection was completed, the data were analyzed with SPSS17.0. Normally distributed data are expressed as χ ± *s*, non-normal distributed data are expressed as median (25th percentile and 75th percentile), and count data are expressed as numerical values and percentages. The *t*-test and *χ*^2^ test were used to compare differences between the two groups. *P* < 0.05 was considered to indicate statistical significance.

## Results

From August 2017 to October 2019, a total of 516 preterm infants met the inclusion criteria: 212 were later excluded, and only 149 preterm infants remained in the DM group and 155 preterm infants remained in the formula group. There were no significant differences in asphyxia (Apgar score at 5 min, <7), gestational age, gender, birth head circumference, birth weight, or prenatal steroid use (*P* > 0.05) (Table 1). None of the values were significantly different between the two groups (Table 1).

**Table 1:** Demographic and clinical parameters between the two groups

Measurement	PF group (*n* = 155)	DM group (*n* = 149)	*P*
Male sex	84	81	0.976
Birth weight (g)	1,255 ± 329	1,239 ± 356	0.684
Birth circumference (cm)	28.9 ± 2.3	29.1 ± 1.9	0.410
Gestational age (week)	30 ± 1.8	30.2 ± 1.6	0.307
Apgar score <7 at 5 min	31	22	0.229
Prenatal steroid use	122	126	0.188

DM: donor milk; PF: preterm formula.

In terms of growth (Table 2), the average daily weight gain and weekly increase in head circumference of the children in the DM group were not statistically different from those in the PF group, which were 17.9 ± 3.5 g versus 18.5 ± 2.7 g, 0.8 ± 0.4 cm versus 0.9 ± 0.3 cm, respectively (*P* > 0.05).

**Table 2:** Growth between the two groups of children

Measurement	PF group	DM group	*P*
Discharge weight (g)	2325 ± 142	2304 ± 131	0.181
Average daily weight gain (g/d)	18.5 ± 2.7	17.9 ± 3.5	0.095
Head circumference at discharge (cm)	32.3 ± 0.8	32.1 ± 1.0	0.054
Weekly increase in head circumference (cm/week)	0.9 ± 0.3	0.8 ± 0.4	0.217

DM: donor milk; PF: preterm formula.

In terms of feeding (Table 3), the feeding intolerance time, duration of parenteral nutrition, and hospitalization time in the DM group were significantly shorter than those in the PF group (*P* < 0.05). In particular, total enteral feeding was achieved 6.7 days earlier in the DM group than it was in the PF group.

**Table 3:** Feeding parameters between the two groups

Measurement	PF group	DM group	*P*
Feeding intolerance time (d)	8.6 ± 2.9	5.2 ± 2.5	<0.001
Duration of parenteral nutrition(d)	21.3 ± 2.4	14.6 ± 3.7	<0.001
Duration of stay at the NICU (d)	39.8 ± 9.3	31.5 ± 8.7	<0.001

DM: donor milk; PF: preterm formula.

**Fig. 1:**
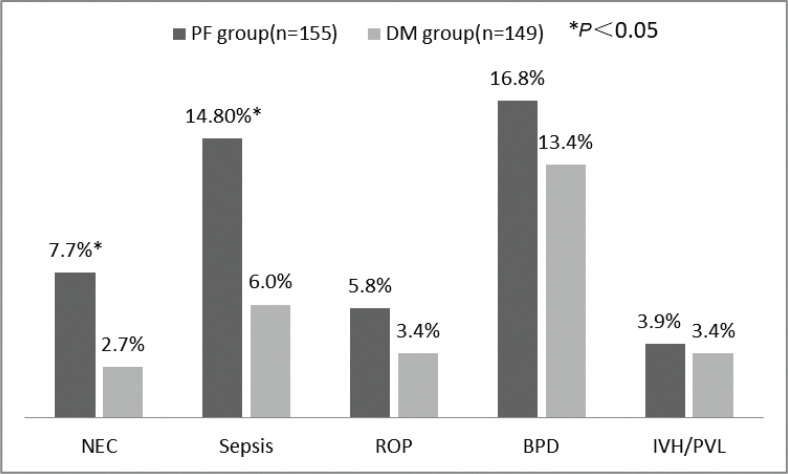
Severe morbidity in study infants at discharge. The morbidities including NEC, sepsis, ROP, BPD, and IVH/PVL between PF group and DM group were compared. The total number in PF group was 155 cases and that in DM group was 149 cases. *P < 0.05. NEC: necrotizing enterocolitis; ROP: retinopathy of prematurity; BPD: bronchopulmonary dysplasia; IVH: intraventricular hemorrhage; PVL: periventricular leukomalacia.

In terms of severe morbidity (Fig. 1), the incidence of NEC and sepsis in the DM group was significantly lower than that in the PF group, which were 2.7% versus 7.7%, 6.0% versus 14.8%, *P* < 0.05, but the incidence of ROP, BPD, and severe IVH was not significantly different between the two groups (*P* > 0.05).

## Discussion

Milk bank is a public institution established for the purpose of collecting, testing, screening, processing, storing, and rational distribution of DM. At present, there are approximately 500 regular milk banks from 27 countries in the world, mainly distributed in Australia, the United States, the United Kingdom, Brazil, Germany, Norway, and other countries.

The present study reported the benefits and disadvantages of preterm DM in China. As China only recently established its first non-profit milk bank for storage of DM exclusivity from preterm infant mothers, the findings of this study make an important contribution to understanding the effects of DM in VLBW infants.

The findings of the present study showed that DM did not have a significant effect on the average daily weight gain and weekly head circumference growth. However, Madore LS et al. (8) showed that a predominantly DM diet in preterm infants could impair early weight gain in the hospital and cause cognitive delays at the age of 1 and 2 years. Their findings indicate that pasteurization may affect macronutrients and enzyme activity and thus result in slow growth. One of the reasons for the difference in the results could be that their DM comes from milk banks, most of which contain full-term human milk and mature milk, while our DM comes from mothers of preterm infants who were hospitalized during the same period, especially VLBW infants, and was used as soon as possible after pasteurization. The composition of human milk from preterm delivery mother differs from that from full-term delivery mother, because preterm milk has higher concentrations of protein, lipid, energy, and immunoglobulin (9). Therefore, the quantity and quality of nutrients of preterm DM are better than that of full-term DM. This might explain why the growth rate of preterm infants fed with pooled full-term DM is slow. Marina showed that pasteurization did not significantly reduce the macro-nutrients and energy content of DM, but slower milk infusion rates significantly impacted the final delivery of fat and energy (10). All of these findings imply that the screening of DM is the key: The mothers of preterm infants should be prioritized as donors. Further, analysis of DM should be performed, if possible, and high-quality milk sources with higher protein content and energy should be selected. Another solution, according to the recommendations of the American Academy of Pediatrics and the Chinese Academy of Pediatrics, is the reasonable fortification of milk to meet the protein and mineral needs of VLBW infants. Early fortification appears to positively affect growth in the absence of any adverse effects, as intensive breastfeeding and PF-fed preterm infants have similar growth rates during hospitalization and can reach normal in utero fetal growth rates (11, 12). Additionally, further research should be conducted on processing DM through methods such as ultra-pasteurization or non-thermal methods, as this could further reduce the loss of fat and nutrients (13).

In this study, the hospitalization period in the DM group was significantly shorter than that in the PF group. Therefore, the use of DM might reduce hospitalization costs. Further, total enteral nutrition was achieved significantly sooner with DM than with PF. This finding is probably related to the beneficial components of human milk that are known to persist in DM even after pasteurization (2). These components include amylase, lipase, protease, growth hormones, and nutrients that promote digestion, immune tolerance, and maturation of gastrointestinal cells, among other functions. These factors also help in absorption and reduce the incidence of feeding intolerance, and this explains why total enteral feeding in the DM group was reached 6.7 days before it was reached in the PF group.

NEC is one of the most serious gastrointestinal diseases in newborn, which mainly occurs in premature infants with immature intestinal tract and imperfect intestinal mucosal barrier. It is especially a serious threat to VLBW infants; it can lead to systemic inflammatory response syndrome and can even be life-threatening. Fortified human milk may interfere with gastric emptying and bowel movements, but does not increase the risk of NEC (14). Previous randomized trials (RTs) and meta-analyses have provided evidence of the relative advantages of human milk over formula; these studies have shown that breastfeeding, whether pasteurized DM or MOM, can prevent NEC and infections and is highly beneficial for VLBW infants (15, 16). In a randomized trial on extremely premature infants, when their owe mother’s milk supply was inadequate, these infants were given either DM and human milk-based human milk fortifier, or PF, reported that the incidence of NEC, as well as surgical NEC, was significantly higher with PF than with DM (17). Our findings are in agreement with this previous study, as DM was found to significantly reduce the incidence of NEC and sepsis and confer protection against infection (Fig. 1). The high osmotic pressure of PF may damage the intestinal mucosa. Although the precise underlying mechanism is unclear, existing evidence shows that oligosaccharides, growth factors, glutamine, l-arginine, lactoferrin, probiotics, and prebiotics contained in human milk can enhance colonization of intestinal symbiotic bacteria, inhibit the growth of pathogenic bacteria, maintain the integrity of the intestinal mucosa, and enhance immunity, thereby reducing the risk of sepsis and NEC. Numerous studies (18, 19) have shown that the duration of PICC or UVC is associated with bloodstream infections. In our study, the duration of central venous catheter placement was shorter in the DM group than that in the PF group; this might have reduced infection in the former.

In contrast to the present findings, recently, a large multi-center, double-blind randomized clinical trial in the Netherlands reported that DM did not have a protective effect against infection and NEC (20). However, in their study, DM was provided only in the first 10 days after birth, and the proportion of human milk in the DM and PF groups was very high (89.1 and 84.5% respectively). This might have led to a serious bias in the comparison analysis. Nonetheless, mounting evidence clearly shows that premature human milk has advantages that no formula can match, and that the benefit for premature infants is directly related to the proportion of human milk consumed (21).

Previous studies have shown that DM can reduce the incidence of BPD (22) and ROP (23), but this study did not find a significant difference between the two groups. This might be related to recent improvements in the formula feed and a lack of research on the topic. Therefore, further large-scale research is needed on the delayed effects of formula.

### Limitations

Our research also had some limitations. The study was observational in nature and was not double-blinded and also only a single-center cohort study with a sample of only 304. As a result, there could have been confounders that affected the validity of the results. Further, the long-term effect of DM and PF on long-term neural development, physical growth, mental development, etc., was not examined.

## Conclusions

We have clearly demonstrated that preterm DM has more protective effect than formula in VLBW infants, who showed improved feeding tolerance, a lower incidence of NEC and sepsis, and a shorter length of hospitalization, and no sign of growth retardation. Although these findings are promising, future research on the long-term impact of this feed is important to confirm these findings.

## Statement

Although present data from this study and many others are finding advantageous in several aspects of DM over PF, however, MOM has incomparable superiority over DM in every aspect. The European Society of Paediatric Gastroenterology, Hepatology and Nutrition Committee on Nutrition recommends the following: The preferred food for premature infants is fortified human milk from MOM (24), and only if there is insufficient MOM or the mother cannot provide breastfeeding, DM becomes the second choice (25).

## References

[1] Corpeleijn WE, Kouwenhoven SM, Paap MC, van Vliet I, Scheerder I, Muizer Y, et al. Intake of own mother’s milk during the first days of life is associated with decreased morbidity and mortality in very low birth weight infants during the first 60 days of life. Neonatology 2012; 102(4): 276–81. doi: 10.1159/000341335 22922675

[2] Lewis ED, Richard C, Larsen BM, Field CJ. The importance of human milk for immunity in preterm infants. Clin Perinatol 2017; 44(1): 23–47. doi: 10.1016/j.clp.2016.11.008 28159208

[3] Miller J, Tonkin E, Damarell RA, McPhee AJ, Suganuma M, Suganuma H, et al. A systematic review and meta-analysis of human milk feeding and morbidity in very low birth weight infants. Nutrients 2018; 10(6): 707. doi: 10.3390/nu10060707 PMC6024377 29857555

[4] Collado MC, Cernada M, Neu J, Perez-Martinez G, Gormaz M, Vento M. Factors influencing gastrointestinal tract and microbiota immune interaction in preterm infants. Pediatr Res 2015; 77(6): 726–31. doi: 10.1038/pr.2015.54 25760550

[5] Committee on Nutrition, Section on Breastfeeding, Committee on Fetus and Newborn. Donor human milk for the high-risk infant: preparation, safety, and usage options in the United States. Pediatrics 2017; 139(1): e20163440. doi: 10.1542/peds.2016-3440 27994111

[6] ESPGHAN Committee in Nutrition, Arslanoglu S, Corpeleijn W, Moro G, Braegger C, Campoy C, et al. Donor human milk for preterm infants: current evidence and research directions. J Pediatr Gastroenterol Nutr 2013; 57(4): 535–42. doi: 10.1097/MPG.0b013e3182a3af0a 24084373

[7] Group of Human Milk Bank CoCHCMDA. [Characteristics of the Chinese human milk banks’ operation]. Zhonghua Er Ke Za Zhi 2017; 55(8): 597–601. doi: 10.3760/cma.j.issn.0578-1310.2017.08.010 28822435

[8] Madore LS, Bora S, Erdei C, Jumani T, Dengos AR, Sen S. Effects of donor breastmilk feeding on growth and early neurodevelopmental outcomes in preterm infants: an observational study. Clin Therapeut 2017; 39(6): 1210–20. doi: 10.1016/j.clinthera.2017.05.341 28576299

[9] Radmacher PG, Lewis SL, Adamkin DH. Individualizing fortification of human milk using real time human milk analysis. J Neonatal Perinatal Med 2013; 6(4): 319–23. doi: 10.3233/NPM-1373113 24441088

[10] Castro M, Asbury M, Shama S, Stone D, Yoon EW, O’Connor DL, et al. Energy and fat intake for preterm infants fed donor milk is significantly impacted by enteral feeding method. JPEN J Parenteral Enteral Nnutr 2019; 43(1): 162–5. doi: 10.1002/jpen.1430 30070721

[11] Adhisivam B, Kohat D, Tanigasalam V, Bhat V, Plakkal N, Palanivel C. Does fortification of pasteurized donor human milk increase the incidence of necrotizing enterocolitis among preterm neonates? A randomized controlled trial. J Mater Fetal Neonatal Med 2019; 32(19): 3232–7. doi: 10.1080/14767058.2018.1461828 29618272

[12] Huston R, Lee M, Rider E, Stawarz M, Hedstrom D, Pence M, et al. Early fortification of enteral feedings for infants <1250 grams birth weight receiving a human milk diet including human milk based fortifier. J Neonatal Perinatal Med 2020; 13(2): 215–21. doi: 10.3233/NPM-190300 31707377 PMC7369034

[13] de Halleux V, Pieltain C, Senterre T, Rigo J. Use of donor milk in the neonatal intensive care unit. Semin Fetal Neonatal Med 2017; 22(1): 23–9. doi: 10.1016/j.siny.2016.08.003 27649995

[14] Brown JV, Embleton ND, Harding JE, McGuire W. Multi-nutrient fortification of human milk for preterm infants. Cochrane Database Syst Rev 2016; (5): CD000343. doi: 10.1002/14651858.CD000343.pub3 27155888

[15] Kantorowska A, Wei JC, Cohen RS, Lawrence RA, Gould JB, Lee HC. Impact of donor milk availability on breast milk use and necrotizing enterocolitis rates. Pediatrics 2016; 137(3): e20153123. doi: 10.1542/peds.2015-3123 26908696 PMC4771129

[16] Quigley M, Embleton ND, McGuire W. Formula versus donor breast milk for feeding preterm or low birth weight infants. Cochrane Database Syst Rev 2018; 6: CD002971. doi: 10.1002/14651858.CD002971.pub4 29926476 PMC6513381

[17] Cristofalo EA, Schanler RJ, Blanco CL, Sullivan S, Trawoeger R, Kiechl-Kohlendorfer U, et al. Randomized trial of exclusive human milk versus preterm formula diets in extremely premature infants. J Pediatr 2013; 163(6): 1592–5.e1. doi: 10.1016/j.jpeds.2013.07.011 23968744

[18] Dubbink-Verheij GH, Bekker V, Pelsma ICM, van Zwet EW, Smits-Wintjens V, Steggerda SJ, et al. Bloodstream infection incidence of different central venous catheters in neonates: a descriptive cohort study. Front Pediatr 2017; 5: 142. doi: 10.3389/fped.2017.00142 28676849 PMC5477168

[19] Milstone AM, Reich NG, Advani S, Yuan G, Bryant K, Coffin SE, et al. Catheter dwell time and CLABSIs in neonates with PICCs: a multicenter cohort study. Pediatrics 2013; 132(6): e1609–15. doi: 10.1542/peds.2013-1645 24218474 PMC3838533

[20] Corpeleijn WE, de Waard M, Christmann V, van Goudoever JB, Jansen-van der Weide MC, Kooi EM, et al. Effect of donor milk on severe infections and mortality in very low-birth-weight infants: the early nutrition study randomized clinical trial. JAMA Pediatr 2016; 170(7): 654–61. doi: 10.1001/jamapediatrics.2016.0183 27135598

[21] Meinzen-Derr J, Poindexter B, Wrage L, Morrow AL, Stoll B, Donovan EF. Role of human milk in extremely low birth weight infants’ risk of necrotizing enterocolitis or death. J Perinatol 2009; 29(1): 57–62. doi: 10.1038/jp.2008.117 18716628 PMC2801431

[22] Villamor-Martinez E, Pierro M, Cavallaro G, Mosca F, Kramer BW, Villamor E. Donor human milk protects against bronchopulmonary dysplasia: a systematic review and meta-analysis. Nutrients 2018; 10(2): 238. doi: 10.3390/nu10020238 PMC5852814 29461479

[23] Kreissl A, Sauerzapf E, Repa A, Binder C, Thanhaeuser M, Jilma B, et al. Starting enteral nutrition with preterm single donor milk instead of formula affects time to full enteral feeding in very low birthweight infants. Acta Paediatr 2017; 106(9): 1460–7. doi: 10.1111/apa.13914 28498519

[24] Agostoni C, Buonocore G, Carnielli VP, Curtis MD, Darmaun D, Decsi T, et al. Enteral nutrient supply for preterm infants: commentary from the European Society of Paediatric Gastroenterology, Hepatology and Nutrition Committee on Nutrition. J Pediatr Gastroenterol Nutr 2010 1; 50(1): 85–91. doi: 10.1097/MPG.0b013e3181adaee0 19881390

[25] Dutta S, Singh B, Chessell L, Wilson J, Janes M, McDonald M, et al. Guidelines for feeding very low birth weight infants. Nutrients 2015 1 8; 7(1): 423–42. doi: 10.3390/nu7010423 25580815 PMC4303848

